# Agreement between a swept-source optical coherence tomography and dual Scheimpflug Placido analyser in healthy, keratoconus suspect and keratoconus eyes

**DOI:** 10.1186/s12886-026-04730-y

**Published:** 2026-03-28

**Authors:** Mohd-Asyraaf Abdul-Kadir, Muhammad Adri Mohamed Shafit, Adzura Salam, Khairidzan Mohd Kamal

**Affiliations:** 1 Department of Ophthalmology, Faculty of Health and Medical Science, Universiti Malaysia Sarawak, Kota Samarahan, Malaysia; 2 Department of Ophthalmology, Kulliyyah of Medicine, International Islamic University Malaysia, Kuala Lumpur, Malaysia; 3 Department of Ophthalmology, Faculty of Medicine, Universiti Sultan Zainal, Abidin Medical Campus, Jalan Sultan Mahmud, 20400 Kuala Terengganu, Terengganu Darul Iman, Malaysia

**Keywords:** ANTERION, GALILEI, Keratoconus, Keratoconus suspect, Agreement, Keratometry, Best fit sphere, Pachymetry

## Abstract

**Purpose:**

To evaluate the agreement and interchangeability of anterior and posterior corneal parameters measured by the swept-source optical coherence tomography–based ANTERION and the dual Scheimpflug–Placido GALILEI G4 in normal, keratoconus suspect (KS), and keratoconus (KC) eyes.

**Methods:**

In this cross-sectional study, 221 eyes (129 normal, 40 KS, 52 KC) were examined using ANTERION and GALILEI G4 in a single session. Anterior and posterior keratometry (K1, K2, K Max), best-fit sphere (BFS), central corneal thickness (CCT), and minimum corneal thickness (MCT) were analysed. Inter-device agreement was assessed using paired statistical tests and Bland–Altman analysis.

**Results:**

In normal eyes, most parameters showed statistically significant mean differences with wide limits of agreement (LoA). In KS eyes, no significant differences were observed in each parameter. In KC eyes, mean differences were minimal, though LoAs were wide, particularly for anterior keratometry and pachymetry; only MCT showed a statistically significant difference. Pachymetric measurements consistently differed between devices, with ANTERION generally reporting smaller values.

**Conclusion:**

The wide LoAs between ANTERION and GALILEI indicate that the devices are not interchangeable, particularly for longitudinal monitoring in keratoconus. Consistent use of a single device is recommended in clinical practice.

## Background

KC has traditionally been described as a noninflammatory, progressive thinning and bulging of the cornea [1]. It typically begins in early adulthood and may cause debilitating visual impairment [2]. In corneal refractive surgery, the early detection of KC has become a crucial practice to prevent possible postoperative ectasia [3, 4]. Furthermore, early detection could halt the progressive loss of vision in KC by performing corneal cross-linking [4]. Advances in diagnostic algorithms utilising the corneal topographic and tomographic assessment have greatly improved the ability to detect KC at an earlier stage [5–9].

Although many devices are available, there is no gold standard tool to determine the clinical accuracy of corneal measurements. Early detection of KC leads to timely intervention, improves patients’ outcomes, and minimises the need for corneal transplantation. No single metric can unequivocally distinguish emerging KC disease from normal corneal data. Corneal topography is a non-contact imaging technique that maps the anterior corneal surface. However, it is unable to provide a complete pachymetry evaluation without the information on the posterior corneal surface. Conversely, corneal tomography studies the whole cornea by obtaining data from the anterior and posterior corneal surfaces allowing three-dimensional reconstruction of the anterior segment. Thus, corneal tomography is the most used technique to diagnose KC as it evaluates the posterior corneal elevation abnormalities even in the subclinical stage [10].

Among currently available imaging modalities, ANTERION is one the newer diagnostic devices. It utilises 1300-nm wavelength infrared light with high scanning speeds [50,000 A-scans/second], thereby provides detailed cross-sectional imaging of anterior segment morphologies. This technology allows superior penetration through opaque media and enables comprehensive analysis of layers of cornea [11]. In addition, the reduced data acquisition time in OCT aids to minimise motion artefacts [10]. In contrast, the GALILEI system employs dual rotating Scheimpflug cameras combined with Placido disc topography to measure corneal elevation and curvature data. The dual cameras corrects decentration errors resulting in more robust corneal topography assessments, and effective surface analysis [12].

The newer ANTERION has been demonstrated to have good intra-session repeatability in measurements of keratometry parameters such as Sim-K, astigmatism magnitude, anterior chamber depth and axial length [13–15]. Few studies demonstrated that ANTERION is an accurate and highly reproducible method for the evaluation of the anterior segment [13, 16–19]. Moreover, it has also been shown to be precise in evaluating cross-sectional pachymetry with wide corneal coverage and can distinguish patients with clinical KC from normal populations [20]. Kim et al. have demonstrated ANTERION effectively differentiates KC from KS and normal corneas by using BFS and BFT-based elevation maps and measuring key parameters like anterior/posterior elevation, pachymetry, and keratometry [21].

GALILEI dual Scheimpflug system is one of the tomography devices for refractive and cataract surgery. The device has been used to assess the effectiveness of collagen crosslinking for KC [22], to measure keratometry and corneal power [23] and to measure central corneal thickness [24]. The GALILEI has also been compared to other devices for predicting residual astigmatism before cataract surgery [25]. Additionally, GALILEI has been found to have high repeatability in most ocular biometric measurements and have a very high agreement with a partial coherence interferometry device, IOL Master 500 [26]. Based on the current data, ANTERION and GALILEI are effective devices for measuring various parameters related to the cornea and anterior segment of the eye.

The comparison among machines to assess their agreement is a way of assessing the exchangeability of measurements captured by different machines and can be an indirect indicator of accuracy. As they both have significant differences in terms of the hardware and software, the study enriches the knowledge of the comparability of new devices to assess their clinical performance. Therefore, comparing these two machines in measuring corneal parameters will evaluate the newer ANTERION against the well-established GALILEI and whether they are interchangeable across different spectra of corneal morphologies.

## Methods

This was a cross-sectional study and was conducted at IIUM Eye Specialist Clinic [IESC], Kulliyyah of Medicine, Kuantan, Pahang from July 2023 to February 2025. The study was approved by IIUM Research Ethics Committee (IREC 2023 − 201). General inclusion criteria include:

- Participants above 18 years of age until 40 years old.
- Participants who can undergo clinical and diagnostic examinations in one session.
- Participants who can fixate on the light in the device and follow instructions.
- Participants who can open their eyes sufficiently to enable a full image area.

Inclusion criteria for healthy eyes group are based on [21]:

- Normal cornea on slit-lamp examination **AND**
- Normal topography and tomography findings; no irregular corneal pattern and no localised steepening.

Inclusion criteria for the KS group were adapted from Martinez-Abad and Pinero [27].

- Fellow normal eyes with unilateral KC **OR**.
- Eyes without clinical KC that demonstrated tomographic findings; one OR more.
  - Abnormal localised steepening.
  - Asymmetric bow tie pattern.
  - Oblique cylinder > 1.50 D.
- Minimum corneal thickness (MCT) > 300 μm.

Inclusion criteria for the KC group were diagnosed based on [21]. The KC was graded according to the Amsler-Krumeich (AK) classification [28].

- Abnormal corneal on slit-lamp examination, one or more of the following:
  - Munson’s sign.
  - Fleischer ring.
  - Vogt striae.
  - Rizzutti sign.
  - Apical thinning.
- **AND** Topography findings.
  - An inferior steepening area.
  - Skewed asymmetric bowtie.
- MCT > 300 μm.

Exclusion criteria include:

- Participants who cannot fixate on the target light in the device during the examination.
- Participants with active ocular surface infection or inflammation (i.e. conjunctivitis, keratitis, uveitis).
- Participants with corneal ectasia besides KC, e.g. pellucid marginal degeneration or keratoglobus.
- Participants with previous acute corneal hydrops or corneal scarring or opacity.
- Participants with a history of corneal surgery, i.e. corneal collagen cross-linking, corneal ring implantation, lamellar surgery or penetrating keratoplasty.
- Participants who wear soft contact lenses within 2 weeks or rigid contact lenses within 4 weeks of involvement in the study.
- Participants with corneal astigmatism greater than 3.00 D (except in the KC patients).

### Data collection method

All participants were recruited according to inclusion and exclusion criteria and underwent assessments using ANTERION and GALILEI G4. Each device was calibrated each day before the first eye is scanned. To minimise tear film disturbance, no eye drop was instilled into each eye before the examination. All scans were performed by a well-trained investigator as per the manufacturer’s instructions. Each eye was scanned first with both devices in the same session; either starting with the GALILEI then ANTERION or vice versa. To ensure the highest physiological stability of the eyes, all measurements will be done at least 3 h after the subject is awake [29]. All measurements were performed within a 15-minute window to minimise each subject’s diurnal variation. For ANTERION, the “Cornea App” scan mode was to measure corneal tomography with 65 radial B-scans consisting of 256 A-scans resulting in an overall amount of 16,640 A-scans covering an 8 mm zone. Only scans of sufficient quality according to the quality-scoring metrics of each device were included in the analysis. Only reading from one eye per participant was included in the study. The one eye was selected randomly using Microsoft Excel to generate randomised numbers (0 = Right, 1 = Left) using the “RAND” formula. All participants completed ocular examination including intraocular pressure measurement using the air puff tonometry and slit lamp biomicroscopy and fundus examination.

### Statistical analysis

Statistical analysis was conducted using the SPSS version 27.0 (SPSS Inc., Chicago, IL, USA). Demographic data was presented as descriptive statistics. The Kolmogorov-Smirnov or Shapiro-Wilk test was used to determine the normality of the data. Paired-T and Wilcoxon tests were used to compare the two instruments’ variables when applicable. The inter-device agreement will be evaluated using the Bland-Altman analysis; the mean difference and 95% limits of agreement (LoA) will be determined for each parameter, and Bland-Altman graphs will be produced. LoAs are derived from the mean difference plus or minus 1.96 times the standard deviation of differences. Clinically acceptable limits for each parameter were previously described by multiple studies [30, 31]. A difference between two devices for any parameter was considered as significant if it would change the refractive outcome by 0.25 D or more. Thus, the value of 0.25 D was chosen as it is the smallest difference in spherical power used in subjective refraction [31]. McLintock et al. previous study also demonstrated clinically accepted limits for pachymetry mean differences was *< +* 10 μm as this was unlikely to cause > 0.01 D change in refractive outcome [31, 32]. A *p*-value of < 0.05 is considered statistically significant.

## Results

### Demographic data

A total of 221 patients were recruited in the study; 129 (Normal), 40 (KS) and 52 (KC) eyes were included. The mean age of participants in each group were 28.5 *±* 7.2 (Normal), 26.8 ± 5.8 (KS) and 22.4 *±* 7.2 (KC) in years. In the KC group, 14 eyes were in Grade 1, 26 eyes were Grade 2, 7 eyes were Grade 3 and 5 eyes were Grade 4 as illustrated in Table 1.

**Table 1:** Demographic profile of participants in each group of normal, KS and KC eyes

Group	*n*	Mean age *±* SD (years)	Gender		Laterality	
			M	F	*R*	L
Normal	129	28.5 *±* 7.2	65	64	64	65
KS	40	26.8 *±* 5.8	22	18	20	20
KC	52	22.4 *±* 7.2	38	14	26	26

### Normal eyes

**Fig. 1:**
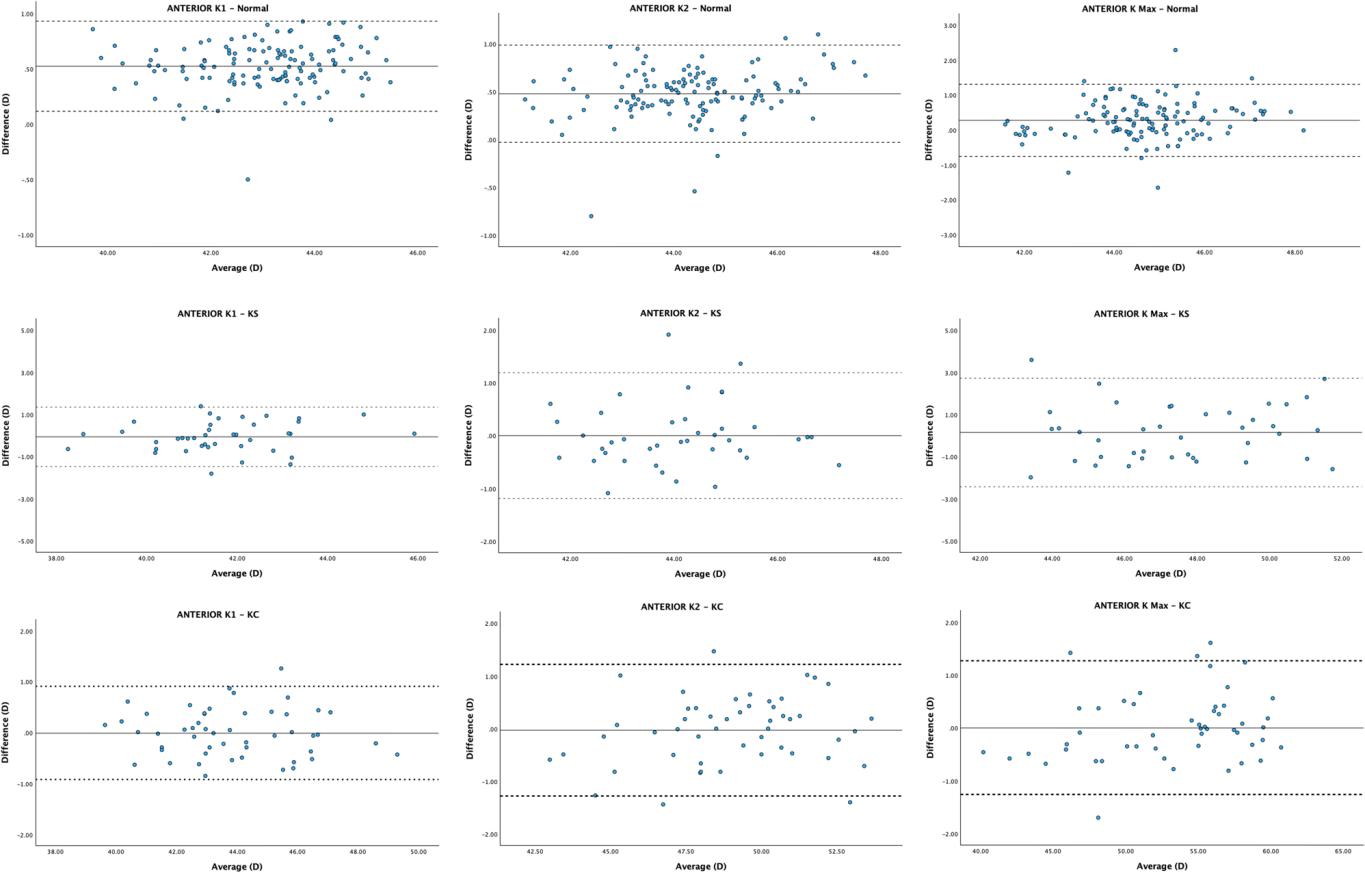
Bland-Altman plots visualising agreement between anterior corneal parameters values of ANTERION and GALILEI. The solid line represents the mean difference, whereas the dotted lines on each side show the upper and lower 95% LoAs

**Fig. 2:**
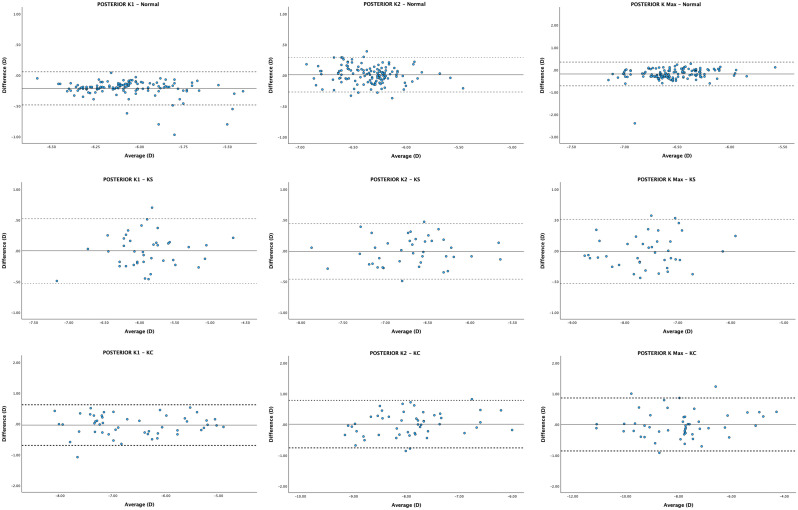
Bland-Altman plots visualising agreement between posterior corneal parameters values of ANTERION and GALILEI. The solid line represents the mean difference, whereas the dotted lines on each side show the upper and lower 95% LoAs

**Fig. 3:**
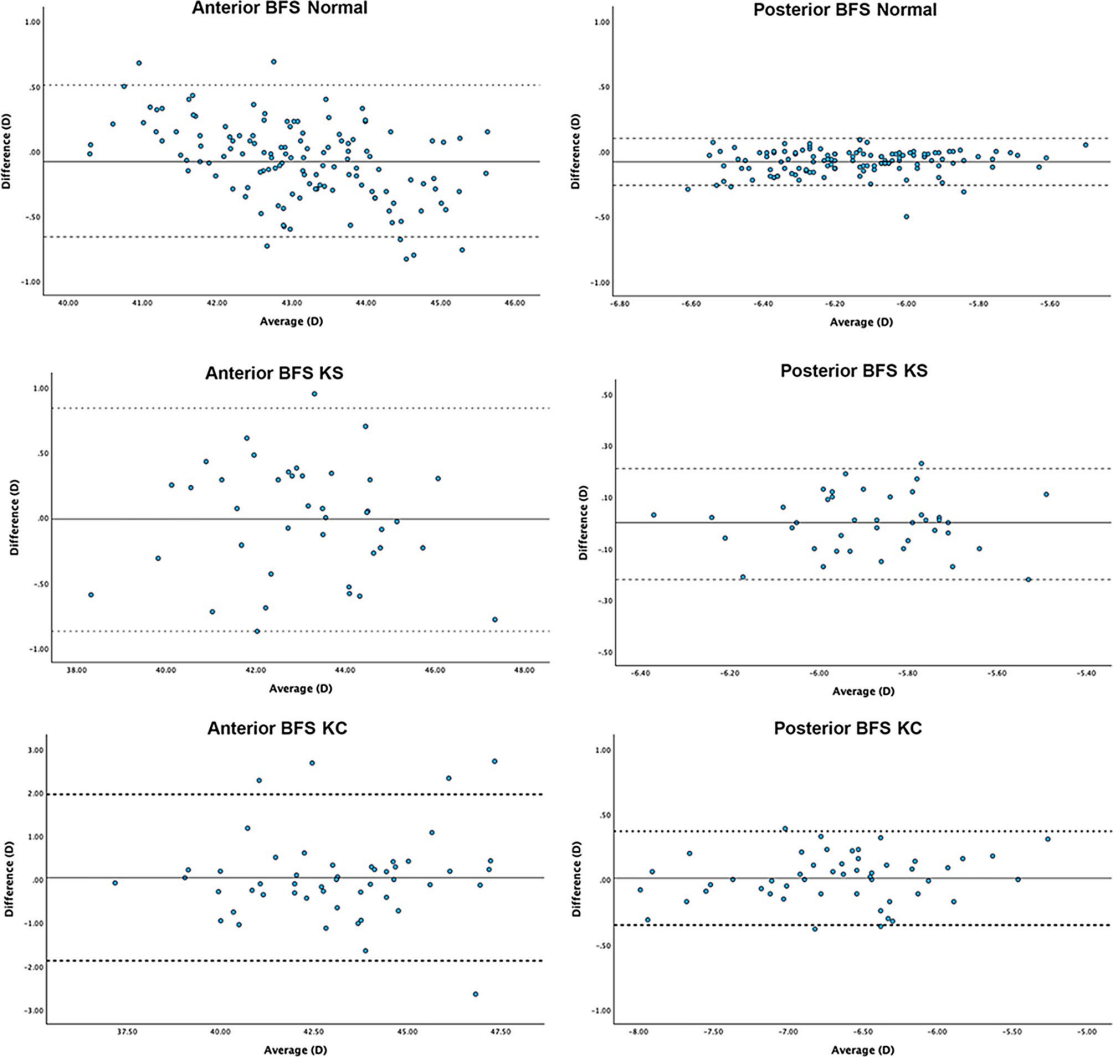
Bland-Altman plots visualising agreement between best-fit spheres values of ANTERION and GALILEI. The solid line represents the mean difference, whereas the dotted lines on each side show the upper and lower 95% LoAs

**Fig. 4:**
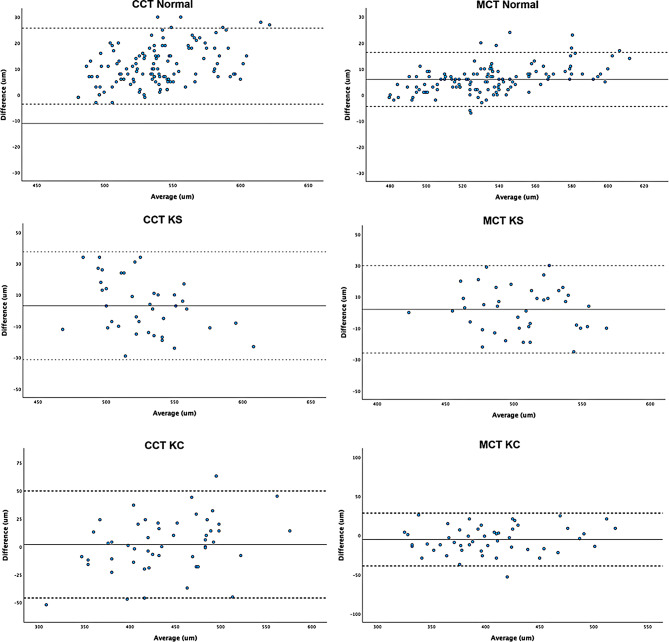
Bland-Altman plots visualising agreement between central and minimal corneal values of ANTERION and GALILEI. The solid line represents the mean difference, whereas the dotted lines on each side show the upper and lower 95% LoAs

The mean difference (MD) and lower and upper LoAs were 0.53 D (0.12, 0.93), 0.48 D (-0.03, 0.99), 0.28 D (-0.75, 1.31), and − 0.08 D (-0.66, 0.51) for anterior K1, K2, K Max and BFS. For posterior corneal curvatures, the MD and lower and upper LoAs were − 0.21 D (-0.48, 0.06), 0.01 D (-0.27, 0.29), -0.19 D (-0.72, 0.35) and − 0.08 D (-0.26, 0.10) for posterior K1, K2, K Max and BFS. For corneal pachymetry, mean difference and lower and upper LoAs for CCT and MCT were − 11.08 μm [-25.75, 3.60] and − 5.92 μm (-16.30, 4.46). All mean differences (MD) reached significance [*p* < 0.05] except posterior K2 (See Table 2). Bland-Altman plots demonstrated the agreement of each biometric parameter within each group between ANTERION and GALILEI (Figs. 1, 2, 3 and 4).

**Table 2:** Mean (± SD) of biometry metrics of ANTERION and GALILEI and agreement of biometry metrics between ANTERION and GALILEI for normal eyes

	Variable	ANTERION (Mean *±* SD)	GALILEI (Mean *±* SD)	p-value	MD	Lower LoA	Upper LoA
Anterior	K1 (D)	43.30 *±* 1.28	42.77 *±* 1.26	**< 0.001**	0.53 *±* 0.21	0.12	0.93
	K2 (D)	44.57 *±* 1.37	44.09 *±* 1.31	**< 0.001**	0.48 *±* 0.26	-0.03	0.99
	K Max (D)	44.53 *±* 1.41	44.80 *±* 1.41	**< 0.001**	0.28 *±* 0.53	-0.75	1.31
	BFS (D)	43.05 *±* 1.11	43.12 *±* 1.25	**0.01**	-0.08 *±* 0.30	-0.66	0.51
Posterior	K1 (D)	-6.14 *±* 0.23	-6.00 *±* 0.20	**< 0.001**	-0.21 *±* 0.14	-0.48	0.06
	K2 (D)	-6.35 *±* 0.27	-6.34 *±* 0.25	0.34	0.01 *±* 0.14	-0.27	0.29
	K Max (D)	-6.10 *±* 1.30	-6.32 *±* 1.14	**< 0.001**	-0.19 *±* 0.27	-0.72	0.35
	BFS (D)	-6.11 *±* 0.22	-6.19 *±* 0.24	**< 0.001**	-0.08 *±* 0.09	-0.26	0.10
Pachymetry	CCT (µm)	533 *±* 29	544 *±* 32	**< 0.001**	-11.08 *±* 7.49	-25.75	3.60
	MCT (µm)	532 *±* 29	538 *±* 32	**< 0.001**	-5.92 *±* 5.30	-16.30	4.46

MD (ANTERION - GALILEI) and limits of agreement are calculated based on the Bland-Altman analysis. D= dioptre, LoA= limit of agreement

### KS eyes

The mean difference and lower and upper LoAs were − 0.06 D (-1.47, 1.35), 0.00 D (-1.19, 1.19), 0.15 D (-2.43, 2.73), and − 0.01 D (-0.87, 0.84) for anterior K1, K2, K Max and BFS. For posterior corneal curvatures, the mean difference and lower and upper LoAs were 0.00 D (-0.53, 0.52), -0.01 D (-0.46, 0.44), -0.01 D (-0.53, 0.51) and 0.00 D (-0.22, 0.21) for posterior K1, K2, K Max and BFS. For corneal pachymetry, mean difference and lower and upper LoAs for CCT and MCT were − 3.08 μm (-31.33, 37.49) and − 2.03 μm (-25.83, 29.89). None of the parameters reached significance (*p* < 0.05) (See Table 3).

**Table 3:** Mean (± SD) of biometry metrics of ANTERION and GALILEI and agreement of biometry metrics between ANTERION and GALILEI for KS eyes

	Variable	ANTERION (Mean *±* SD)	GALILEI (Mean *±* SD)	p-value	MD	Lower LoA	Upper LoA
Anterior	K1 (D)	41.69 *±* 1.48	41.63 *±* 1.59	0.61	-0.06 *±* 0.72	-1.47	1.35
	K2 (D)	44.06 *±* 1.42	44.06 *±* 1.44	0.99	0.00 *±* 0.61	-1.19	1.19
	K Max (D)	47.40 *±* 2.50	47.56 *±* 2.59	0.46	0.15 *±* 1.32	-2.43	2.73
	BFS (D)	43.06 *±* 1.85	43.04 *±* 1.83	0.90	-0.01 *±* 0.44	-0.87	0.84
Posterior	K1 (D)	-5.89 *±* 0.46	-5.90 *±* 0.49	0.94	0.00 *±* 0.27	-0.53	0.52
	K2 (D)	-6.70 *±* 0.48	-6.70 *±* 0.49	0.82	-0.01 *±* 0.23	-0.46	0.44
	K Max (D)	-7.56 *±* 0.65	-7.60 *±* 0.68	0.79	-0.01 *±* 0.26	-0.53	0.51
	BFS (D)	-5.88 *±* 0.19	-5.89 *±* 0.19	0.93	0.00 *±* 0.11	-0.22	0.21
Pachymetry	CCT (µm)	525 *±* 34	529 *±* 27	0.28	-3.08 *±* 17.56	-31.33	37.49
	MCT (µm)	504 *±* 34	506 *±* 33	0.38	-2.03 *±* 14.22	-25.83	29.89

MD (ANTERION– GALILEI) and limits of agreement are calculated based on the Bland-Altman analysis. D= dioptre, LoA= limit of agreement

### KC eyes

The mean difference and lower and upper LoAs were − 0.01 (-0.92, 0.91), -0.03 (-1.28, 1.22), 0.00 (-1.26, 1.27), and 0.04 (-1.87, 1.96) for anterior K1, K2, K Max and BFS. For posterior corneal curvatures, the mean difference and lower and upper LoAs were − 0.04 (-0.70, 0.62), 0.01 (-0.76, 0.78), 0.00 (-0.86, 0.86) and 0.01 (-0.35, 0.37) for posterior K1, K2, K Max and BFS. For corneal pachymetry, mean difference and lower and upper LoAs for CCT and MCT were 1.90 μm (-45.94, 49.74) and − 5.30 μm (-39.06, 28.46). Only the mean difference in MCT reached significance (*p* < 0.05) (See Table 4).

**Table 4:** Mean (± SD) of biometry metrics of ANTERION and GALILEI and agreement of biometry metrics between ANTERION and GALILEI for KC eyes

	Variables	ANTERION (Mean *±* SD)	GALILEI (Mean *±* SD)	p-value	MD	Lower LoA	Upper LoA
Anterior	K1 (D)	43.83 *±* 2.20	43.84 *±* 2.22	0.91	-0.01 *±* 0.47	-0.92	0.91
	K2 (D)	49.01 *±* 2.67	49.02 *±* 2.57	0.89	-0.03 *±* 0.64	-1.28	1.22
	K Max (D)	53.11 *±* 5.37	53.10 *±* 5.22	0.61	0.00 *±* 0.65	-1.26	1.27
	BFS (D)	43.12 *±* 2.47	43.07 *±* 2.37	0.76	0.04 *±* 0.98	-1.87	1.96
Posterior	K1 (D)	-6.59 *±* 0.90	-6.54 *±* 0.87	0.38	-0.04 *±* 0.34	-0.70	0.62
	K2 (D)	-7.87 *±* 0.81	-7.86 *±* 0.71	0.93	0.01 *±* 0.39	-0.76	0.78
	K Max (D)	-7.91 *±* 1.57	-7.91 *±* 1.51	0.99	0.00 *±* 0.44	-0.86	0.86
	BFS (D)	-6.67 *±* 0.64	-6.68 *±* 0.61	0.66	0.01 *±* 0.18	-0.35	0.37
Pachymetry	CCT (µm)	440 *±* 62	438 *±* 54	0.58	1.90 *±* 24.41	-45.94	49.74
	MCT (µm)	402 *±* 52	407 *±* 49	**0.04**	-5.30 *±* 17.23	-39.06	28.46

MD (ANTERION – GALILEI) and limits of agreement are calculated based on the Bland-Altman analysis. D= dioptre, LoA= limit of agreement

## Discussion

This study assessed the agreement between ANTERION and GALILEI in measuring anterior and posterior corneal parameters, as well as pachymetric values, in normal eyes, KS, and KC eyes. The findings provide insights into the comparability of these two devices across different corneal conditions. Recent studies have demonstrated that while these devices exhibit high individual performance [16, 19, 26], discrepancies in measurements can arise due to systemic biases inherent to different technologies.

In normal eyes, a strong to excellent correlation was observed between ANTERION and GALILEI for corneal keratometry, elevation map and pachymetry. Most of the corneal parameters showed statistically significant differences except for anterior K Max and BFS and posterior K2. However, the mean differences were still within clinically acceptable limits for corneal keratometry and BFS, within *±* 0.25 D except for anterior K1 and K2. Pachymetric LoAs were also slightly exceeded the ideal *±* 10 μm but remained tolerable in clinical practice, provided normal corneal morphologies with diagnostic intention to screen rather than longitudinal monitoring. The wide LoAs for all parameters suggest poor agreement between the devices thus not interchangeable. These findings were also supported by previous studies; significant differences existed between ANTERION and GALILEI G6 in anterior corneal curvatures, corneal power, lens thickness (LT), CCT, anterior chamber depth (ACD) and white-to-white (WTW) [31, 33, 34].

The measurements revealed a systematic difference, with ANTERION consistently reporting lower central and minimum corneal thickness values compared to GALILEI. These systematic differences were likely due to the different technologies by each device to measure pachymetry. These discrepancies are likely due to the imaging wavelengths, acquisition speed and internal data processing algorithms in each device. GALILEI utilised high-resolution elevation and curvature data and the dual-camera setup corrects decentration errors to improve accuracy and repeatability [35]. Meanwhile, ANTERION employs a swept-source OCT technology with longer wavelengths operated at 1.6 MHz A-scan rate and able to penetrate through dense media [33].

For KS eyes, differences in mean values were minimal and did not reach statistical significance for all parameters. Pachymetric measurements exhibited some level of disagreement, with GALILEI reporting slightly higher values. Although the mean differences were small, the wide LoAs render both devices not interchangeable. There are no previous studies done studying the agreement between the two diagnostic devices in this specific group.

Recognising KS eyes is fundamental in preoperative planning for refractive surgery thus minimising the risk of post-LASIK ectasia [36]. Diagnosing KS eyes is challenging due to absence of standardised diagnostic criteria and often, they exhibit subtle differences to normal cornea leading to misdiagnosis and unfavorable complication [37, 38]. Therefore, various studies have investigated different corneal structures to delineate better the features of KS corneas. Kamiya et al. (2014) had shown corneal elevation correlated significantly to the severity of KC and thus highlighting its role in diagnosing KS [39]. Furthermore, the expanded KISA index by incorporating the tomographic changes into the established KISA index was able to reliably classify normal, KS (including subclinical) and manifest KC [40].

In KC eyes, most paramaters between ANTERION and GALILEI demonstrated mean differences close to zero. The wide LoAs for anterior steep K and K Max exceeded *±* 1.25 D, which reflects the greater variability of corneal irregularity in KC. Anterior BFS had fairly wide LoA *±* 1.87 D which was likely due to localised protrusions that influence the BFS assessments. Pachymetry assessments (CCT, MCT) showed very wide LoAs (*±* 39–50 μm) which exceeded the acceptable *±* 10 μm limits for clinical threshold.

Our findings suggested that while most readings remain broadly comparable, the wide LoAs deem both devices should not be used interchangeably in KC follow-up, as inter-device variability could mask or mimic true disease change. Maintaining consistency in device use is essential for accurate longitudinal monitoring, particularly when assessing eligibility for corneal cross-linking. We did not observe statistically significant differences in most mean differences across corneal parameters unlike previous study by Lee et al. This was most likely due to our sample comprised of patients with mild to moderate KC while Lee et al. included more advanced stages of KC [41]. Yusuf et al. commented in their study of 20 patients with KC that there were considerable differences between ANTERION and GALILEI with the most disparity observed in K Max and pachymetry, with the maximal difference 2.1 D and 21 μm [42]. Conversely, Lee et al. (2024) emphasises in KC patients, the reliability of measurements can decrease with the severity of the disease, leading to significant differences in keratometry values between devices SS-OCT and GALILEI. This variability is critical, as it suggests that while devices may perform well individually, their interchangeability in clinical settings is questionable, particularly in patients with ectatic corneas [41]. Furthermore, significant differences were also observed in the measurements among the three Scheimpflug devices in KC group, indicating that they are not interchangeable for anterior segment assessment [43].

Interestingly, while most corneal parameters exhibit statistically significant differences between ANTERION and GALILEI in the normal group, none reached significance in the KS group and only MCT exhibited significant difference in KC group. This is potentially due to the larger sample size in normal group which gives greater power to detect small mean differences compared to the KS and KC groups in our study. Moreover, these differences are likely attributed to the inherent device bias in ANTERION and GALILEI despite good overall correlation and in particular normal cornea with low biological variability. Small systematic measurement biases between imaging systems, even among the same Scheimpflug devices can be statistically identified in normal corneas due to their great regularity and narrow physiological range [44]. We were also using the older GALILEI G4 and although it was identical to current new version GALILEI G6 and deemed no difference between the two models, the updated software and technology of G6 may limit the generalisability of this result [45]. Meanwhile, KC is characterised by severe asymmetry, thickness, and irregularity of the cornea, which leads to a significantly higher dispersion of anterior segment characteristics. This increased variability may outweigh subtle device-related differences, leading to an apparent absence of statistically significant inter-device differences in KC eyes. Previous investigations comparing anterior segment imaging methods in normal and ectatic corneas have revealed similar results, with keratoconus showing higher agreement due to wider parameter ranges rather than true measurement similarity [46–48]. This interpretation is consistent with established principles of agreement analysis, which emphasise that population variability strongly influences the detectability of systematic bias [49]. However, the absence of statistically significant differences in keratoconus eyes should not be interpreted as evidence of interchangeability between devices.

There are limitations to the study. The limited sample size in each group, particularly KC group may undermine the generalisation of the results in the population. Additionally, variations in keratoconus severity within the study group could cause skewing of the results, as different stages of KC may respond differently to each diagnostic tool [50]. Furthermore, we did not repeat measurements for each patient to evaluate the repeatability of the results. Both machines had shown high intra-session repeatability based on previous studies [13–15, 26]. As both machines utilise different technologies; ANTERION measures the surface directly while GALILEI reconstructs the corneal mapping from image intensity and Placido ripples, these differences accounted for the discrepancies of corneal curvatures and pachymetry between the ANTERION and GALILEI [11, 12].

## Conclusion

Overall, this study demonstrates between ANTERION and GALILEI, there were wide LoAs were observed across multiple corneal parameters and morphologic spectra. Accordingly, caution is warranted when interpreting posterior keratometric and pachymetric values, particularly in KC patients, where wide LoA may influence longitudinal assessment. Therefore, the two devices should not be used interchangeably. Clinicians may consider the complimentary use of both systems to optimise patient outcomes in corneal ectasia management and preoperative surgical assessment. Future studies should aim to standardise measurement protocols and establish consensus thresholds for interpreting corneal parameters across different devices to improve clinical outcomes.

## Data Availability

The datasets used and/or analysed during the current study are available from the corresponding author on reasonable request.

## Abbreviations

KC: Keratoconus
KS: Keratoconus suspect
BCVA: Best Corrected Visual Acuity
OCT: Optical Coherence Tomography
D: Dioptre
BFS: Best-Fit Sphere
CCT: Central corneal thickness
MCT: Minimal corneal thickness
